# Integrative microbiome and transcriptome analyses reveal *Telluria* sp. 100-57A as a candidate for aphid suppression in pepper

**DOI:** 10.3389/fpls.2026.1861231

**Published:** 2026-07-06

**Authors:** Jun Su Noh, Gyeongjun Cho, Jihye Jung, Jeong-Seon Kim, Do-Hyun Kim, Jaekyeong Song, Sieun Kim, Jegadeesh Raman, Soo-Jin Kim

**Affiliations:** Division of Agricultural Microbiology, National Institute of Agricultural Sciences, Rural Development Administration, Wanju, Republic of Korea

**Keywords:** biological control, *Capsicum annuum* (Pepper), defense priming, *Myzus persicae* (green peach aphid), rhizosphere microbiome

## Abstract

Management of *Myzus persicae* in pepper still depends largely on insecticides, but resistance limits the long-term effectiveness of chemical control. Here, we used a microbiome-guided approach to identify rhizosphere bacteria associated with aphid infestation and to evaluate their potential for aphid suppression. Pepper plants were grown in soil collected from nine field sites, and rhizosphere bacterial communities were profiled using full-length 16S rRNA gene sequencing. After excluding soils with extreme physicochemical profiles, aphid-infested plants exhibited distinct rhizosphere bacterial communities characterized by the enrichment of *Massilia, Rhizobium, and Chujaibacter*. To further investigate these community-level patterns, culture-dependent isolation from aphid-associated rhizosphere soils yielded five strains assigned to the genera *Telluria* and *Massilia*. Among them, *Telluria* sp. 100-57A consistently reduced the estimated aphid population growth rate by 36.2% and 41.5% at the 500- and 1,000-fold dilutions, respectively, compared with the negative control. This strain also induced credible avoidance of treated leaves in detached-leaf choice assays. Transcriptome analysis of pepper plants treated with strain 100-57A showed enrichment of defense-related, jasmonic acid-related, and wound-response categories during the early response period relative to the negative control. Compared with acibenzolar-S-methyl (ASM), strain 100-57A showed stronger enrichment of photosynthesis-related categories, suggesting a defense-associated transcriptional response distinct from ASM treatment. These findings support microbiome-informed strategies for identifying aphid-suppressive bacteria and highlight *Telluria* sp. 100-57A as a promising candidate for microbiome-based aphid management in pepper.

## 1 Introduction

Pepper *(Capsicum annuum* L.) is an economically important crop widely cultivated for fresh consumption and food processing (Hernández-Pérez et al., 2020). However, pepper production is constrained by several insect pests, including aphids, caterpillars, and thrips, which reduce both yield and fruit quality (Dekebo, 2022). Among the insect pests affecting pepper, the green peach aphid, *Myzus persicae*, is considered one of the most destructive and widely distributed aphid species (Sun et al., 2018). The host spectrum of *M. persicae* encompasses over 400 plant species from a wide variety of families, including *Solanaceae, Brassicaceae*, and *Leguminosae* (Bass et al., 2014). *M. persicae* infestation causes direct damage through phloem sap feeding, resulting in leaf curling, chlorosis, and altered photosynthetic performance (Florencio-Ortiz et al., 2021; Poljaković-Pajnik et al., 2024). The secretion of honeydew by this species also creates favorable conditions for sooty mold development on plant surfaces (Bocco et al., 2021). Beyond direct feeding damage*, M. persicae* contributes to disease spread by transmitting more than 100 plant viruses, leading to substantial declines in crop yield and fruit quality (Dong et al., 2024). Viral infections further weaken plant vigor and can exacerbate plant stress under field conditions (Jiménez et al., 2024).

To reduce aphid-related crop damage, insecticides such as neonicotinoids, pyrethroids, organophosphates, and carbamates have been extensively applied for the management of *M. persicae* (Bass et al., 2014; Umina et al., 2014; de Little and Umina, 2017). However, continuous exposure to insecticides favors the survival of resistant individuals, leading to an increasing prevalence of resistance within aphid populations. Furthermore, *M. persicae* reproduces parthenogenetically and has a short generation time, enabling rapid population expansion and facilitating the spread of resistant genotypes under continuous insecticide exposure (Bass et al., 2014). Accordingly, reduced susceptibility to neonicotinoids, pyrethroids, and carbamates has been reported in field populations worldwide (Bass et al., 2011; Kirkland et al., 2023). In particular, mutations in acetylcholinesterase and voltage-gated sodium channel genes have been associated with reduced insecticide sensitivity in aphid populations (Martinez-Torres et al., 1999; Nabeshima et al., 2003). These target-site resistance mechanisms reduce insecticide efficacy and often result in repeated pesticide applications, increased production costs, environmental concerns, and reduced sustainability of pepper production. Therefore, the development of alternative and environmentally sustainable strategies for aphid management is urgently needed.

Advances in microbiome research have revealed that plant-associated microbial communities play important roles in plant adaptation to biotic stress. In particular, beneficial rhizobacteria can activate induced systemic resistance (ISR) through salicylic acid-, jasmonic acid-, and ethylene-associated defense pathways (Bakker et al., 2013; Pieterse et al., 2014; Jung et al., 2024). Increasing evidence suggests that herbivore infestation can alter plant physiology and root exudation patterns. These changes can reshape rhizosphere microbial communities and promote the recruitment of microorganisms involved in plant defense and stress adaptation (Friman et al., 2021; Sun et al., 2025). Aboveground infestation by whitefly (*Bemisia tabaci*) has also been shown to modify the pepper rhizosphere microbiome, demonstrating that herbivory can influence belowground microbial communities through plant-mediated signaling (Kong et al., 2016). Similar herbivore-induced microbiome shifts have been reported in other plant–insect systems, supporting the idea that plants respond to insect infestation by altering the composition of their associated rhizosphere microbiota (French et al., 2021; Gao et al., 2024; Yang et al., 2025). This concept has also been highlighted in a recent review on beneficial microbe-enriched rhizosphere systems for insect defense (Liu et al., 2024). Together, these findings indicate that herbivore-responsive rhizosphere microorganisms may represent valuable resources for microbiome-assisted pest management.

Previous studies in pepper have shown that aphid infestation can influence belowground microbial recruitment, including the enrichment of beneficial rhizobacteria (Lee et al., 2012). However, it remains unclear how aphid infestation reshapes rhizosphere bacterial community structure and whether aphid-responsive taxa contribute functionally to host defense against *M. persicae*. We hypothesized that aphid infestation would selectively enrich rhizosphere bacterial taxa capable of enhancing host resistance against *M. persicae*. To test this hypothesis, we analyzed rhizosphere bacterial communities of aphid-infested pepper plants using 16S rRNA gene amplicon sequencing, identified aphid-responsive taxa, and isolated candidate bacterial strains from aphid-associated rhizosphere soils. Functional analyses were subsequently conducted to evaluate their effects on aphid performance and defense-associated host responses, with the aim of identifying microbial resources for microbiome-based aphid management in pepper.

## 2 Materials and methods

### 2.1 Insect culture and plant material

The green peach aphid (*Myzus persicae*) was obtained from the insect rearing facility of the Crop Protection Division, National Institute of Agricultural Sciences, Wanju, Korea. Aphids were reared in cages (40 × 40 × 40 cm) on 4-week-old pepper (*Capsicum annuum* cv. ‘Nockgwang’) plants under 24 °C, 60 ± 5% relative humidity, and a 16L:8D light:dark cycle. Pepper plants were grown in bed soil for 4 weeks under tap-water irrigation without additional fertilizer application. For long-term maintenance of the aphid colony, fresh healthy 4-week-old pepper plants were provided weekly.

### 2.2 Experimental design using soils collected from field sites

To identify rhizosphere taxa enriched following aphid infestation, a greenhouse experiment was conducted using field-derived soils collected from nine pepper-growing fields across five regions in Korea: Gunsan (GS; GS1 and GS2), Gunwi (GW; GW1, GW2, and GW3), Hamyang (HY), Jangsu (JS), and Sunchang (SC; SC4 and SC5). At each field, topsoil was collected from multiple points at a depth of 0 to 15 cm and pooled to obtain a site-level composite soil sample. The composite soils were homogenized by site and cleared of visible stones, plant residues, and large roots before pot preparation. Detailed metadata for all sampling sites are provided in **Table 1**. Each pot was filled with approximately 750 g of soil from each sampling site, and one 4-week-old pepper seedling grown in potting substrate was transplanted into each pot. For each soil origin and treatment, 15 independent pots were prepared. The experiment was conducted for 4 weeks from May to June 2024 in a greenhouse divided into two mesh-separated compartments to prevent aphid movement and cross-contamination between treatments. One compartment was designated for aphid infestation, whereas the other served as the untreated control. These compartments were used solely for physical separation and were not considered biological replicates or experimental blocks in the downstream microbiome analysis. Seven days after transplantation, 100 aphids were introduced onto each plant in the aphid-infested treatment, whereas no aphids were applied to the untreated control plants. Plants were monitored for three weeks after aphid infestation under greenhouse conditions. For rhizosphere microbiome analysis, four independent pots were randomly selected from each treatment at each sampling site and used as biological replicates. Plants were carefully collected at harvest, after which bulk soil was removed from the roots by gentle shaking. Soil that remained attached to the root surface was collected as rhizosphere soil. The collected samples were immediately transferred to sterile tubes and stored at −80 °C until DNA extraction.

**Table 1:** Sampling information for the nine field-derived soils used in this study.

Sample ID	Sample site	Latitude	Sample date	Crop
GS1	Gunsan, jeonbuk, Korea	36°39’34”N/127°52’22”E	2023/03/31	Pepper
GS2	Gunsan, jeonbuk, Korea	36°39’47”N/127°52’27”E	2023/03/31	Pepper
GW1	Gunwi, gyeongbuk, Korea	35°34’29”N/127°25’46”E	2023/05/05	Pepper
GW2	Gunwi, gyeongbuk, Korea	35°36’38”N/127°48’34”E	2023/05/06	Pepper
GW3	Gunwi, gyeongbuk, Korea	35°21’23”N/127°04’34”E	2023/05/11	Pepper
HY	Hamyang, gyeongnam, Korea	35°21’31”N/127°04’51”E	2023/05/11	Pepper
JS	Jangsu, jeonbuk, Korea	36°11’17”N/128°33’56”E	2023/05/18	Pepper
SC4	Sunchang, jeonbuk, Korea	36°09’10”N/128°41’10”E	2023/05/18	Pepper
SC5	Sunchang, jeonbuk, Korea	36°09’56”N/128°38’41”E	2023/05/18	Pepper

### 2.3 Soil physicochemical analysis and site grouping

To characterize edaphic heterogeneity among sampling sites, physicochemical properties were measured for the field soils used in this study. A subsample of each field-derived soil was air-dried and passed through a 2 mm sieve before physicochemical analysis. Soil pH and electrical conductivity (EC) were measured in a 1:5 (w/v) suspension prepared with distilled water. The remaining chemical properties were determined at the Korea Agriculture Technology Promotion Agency according to standard soil analysis procedures. These properties included ammonium nitrogen (NH4_N), nitrate nitrogen (NO3_N), total nitrogen (T_N), organic matter (OM), cation exchange capacity (CEC), available phosphate (Av_P2O5), and exchangeable cations. Exchangeable K, Ca, Mg, and Na were expressed as Ex_K, Ex_Ca, Ex_Mg, and Ex_Na, respectively. For downstream analysis, sites were grouped using study-specific operational thresholds to reduce strong edaphic confounding rather than to define universal agronomic critical limits. Sites with pH (1:5)< 5.5, EC (1:5) ≥ 10 dS/m, NH4_N ≥ 40 mg/kg, or NO3_N ≥ 400 mg/kg were assigned to the Excluded group. The remaining sites were assigned to the Primary group for downstream aphid-responsive microbiome analysis. To support this grouping, PCA was performed using soil physicochemical variables. Skewed variables were log1p-transformed, and all variables were standardized by z-score scaling before PCA. A 95% confidence ellipse was calculated from the PCA scores of the Primary group and used to evaluate whether the Excluded sites were separated from the main edaphic space (Worley et al., 2013; Jolliffe and Cadima, 2016).

### 2.4 DNA extraction, full-length 16S rRNA gene sequencing, and data analysis

DNA was extracted from 72 rhizosphere soil samples (nine soil origins × two aphid infestation treatments × four biological replicates) using the FastDNA Spin Kit for Soil (MP Biomedicals, USA). Sodium phosphate (978 μL) and MT buffer (122 μL) were mixed with 500 mg of rhizosphere soil, followed by homogenization using a FastPrep-24 at 6 m/s for 40 s. After homogenization, 250 μL of protein precipitation solution was added to the lysate, and the sample was centrifuged at 14,000 × g for 5 min. The supernatant was collected in a 15 mL tube, mixed thoroughly with 1 mL of binding matrix through inversion for 2 min, and then left undisturbed for 3 min to allow DNA to bind to the matrix. Bound DNA was subsequently recovered by ethanol washing and elution. Full-length 16S rRNA gene (V1–V9) amplicon sequencing on the PacBio platform was conducted by Macrogen (Seoul, Republic of Korea). The extracted metagenomic DNA served as template DNA for amplification with the universal bacterial primers 27F (5′-AGRGTTYGATYMTGGCTCAG-3′) and 1492R (5′-RGYTACCTTGTTACGACTT-3′). PCR consisted of an initial denaturation at 95 °C for 3 min, followed by 25 cycles of 95 °C for 30 s, 57 °C for 30 s, and 72 °C for 60 s. Amplicons were then prepared as SMRTbell libraries for PacBio sequencing. Sequencing data were processed using Macrogen’s internal QIIME 2 pipeline, in which amplicon sequence variants (ASVs) were inferred with DADA2 (Callahan et al., 2016). Per-sample read processing statistics, including input reads, filtered reads, denoised reads, and non-chimeric reads are provided in **Supplementary Table 1**. Rarefaction analysis was performed to evaluate whether sequencing depth was sufficient for downstream diversity analyses, and the rarefaction curves are shown in **Supplementary Figure 1**. Taxonomic assignment of ASVs was performed using BLAST against the NCBI 16S rRNA database downloaded on June 16, 2023. ASV abundance and taxonomic data were analyzed using the R packages phyloseq (version 1.50.0; McMurdie and Holmes, 2013), tidyverse (version 2.0.0; Wickham et al., 2019), and ANCOMBC (version 2.8.1; Lin and Peddada, 2020) to evaluate alpha and beta diversity, relative abundance, and differential abundance.

### 2.5 Isolation and identification of *Telluria* and *Massilia* spp. from rhizosphere soil

For bacterial isolation, rhizosphere soil was serially diluted (10–^3^ to 10^-6^) in sterile distilled water, and aliquots were spread onto Reasoner’s 2A (R2A) agar and water agar plates. Plates were incubated at 28 °C for 2–3 days, and individual colonies were selected and purified. The purified isolates were initially identified by 16S rRNA gene sequencing (Genotech, Daejeon, Korea). Taxonomic assignment of 16S rRNA gene sequences was performed using the EZBioCloud database. The obtained sequences were aligned in BioEdit, and phylogenetic relationships were inferred by the neighbor-joining method in MEGA version 7.0. The reliability of the phylogenetic tree was evaluated by bootstrap analysis with 1,000 replications, and bootstrap values were shown at the nodes of the tree. The identified isolates were grown in R2A broth at 28 °C and 180 rpm for 48 h, mixed with sterile 50% glycerol (1:1, v/v), and stored at −80 °C for subsequent experiments.

### 2.6 Assessment of aphid-suppressive activity of microbial cultures applied by soil drench

Four-week-old pepper plants were used as the experimental host plants. For the microbial treatments, five bacterial strains, including four *Telluria* strains (85-37A, 85-42A, 100-15A, and 100-57A) and one *Massilia* strain (85-53A), were cultured independently in R2A broth at 28 °C and 180 rpm for 3 days. For inoculum standardization, each culture was adjusted to an optical density at 600 nm (OD_600_) of 1.0, corresponding to approximately 1.0 × 10^9^ CFU/mL. The standardized cultures were then diluted 500- and 1,000-fold with sterile distilled water, resulting in final cell densities of approximately 2.0 × 10^6^ and 1.0 × 10^6^ CFU/mL, respectively. Ten milliliters of each diluted culture were applied per pot by soil drenching. The microbial treatments were applied 7 and 3 days before aphid infestation. For the negative control, R2A broth diluted 500- and 1,000-fold with sterile distilled water was applied. The positive control consisted of a commercial formulation containing acibenzolar-S-methyl (ASM; 50% a.i., Syngenta, China), diluted to 0.2 mg a.i./mL. Both treatments were applied at 10 mL per pot. Plants were infested by placing five newly hatched *M. persicae* nymphs on the youngest leaves of each plant. Aphid numbers were recorded daily for 10 days starting the day after infestation, with each treatment replicated five times. The daily population growth rate (r) in response to microbial treatment was estimated using Bayesian generalized linear mixed models fitted with the R package brms. Separate models were fitted for the 500- and 1,000-fold dilution experiments using a negative binomial distribution with a log link. Observation day was included as a continuous predictor, while strain-specific random intercepts and slopes were incorporated into the model. Replicate identity was included as a random intercept. Markov chain Monte Carlo (MCMC) sampling was run with four chains of 20,000 iterations, including 8,000 warm-up iterations per chain, and treatment-specific growth rates were then estimated from the posterior distribution of the day-slope parameter using the following equation:


$$N\left(t\right)=N_{0}\cdot e^{rt}$$

where *N*(*t*) is the aphid population size at time *t*, *N_0_* is the initial population size, *r* is the intrinsic rate of population increase, e is the base of the natural logarithm, and *t* is the observation day.

### 2.7 Assessment of aphid repellency using a detached-leaf assay

Bacterial cultures were prepared as described in Section 2.6. For the detached-leaf assay, cultures were diluted 500-fold with sterile distilled water and supplemented with 0.02% (v/v) Tween 20. Fully expanded leaves were detached from 4-week-old pepper plants, and 3 mL of each diluted microbial suspension was sprayed onto the detached-leaves and allowed to air-dry. For the negative control, R2A broth diluted 500-fold and supplemented with 0.02% Tween 20 was sprayed, whereas the positive control consisted of ASM diluted to 0.2 mg a.i./mL and sprayed at 3 mL per leaf. After drying, treated and untreated detached-leaves were fixed with adhesive stickers and mounted on aluminum foil sheets (7.5 × 7.5 × 1.0 cm) inside rearing Petri dishes (9.5 × 9.5 × 4.0 cm). To reduce leaf desiccation, 2 mL of sterile distilled water was added to each dish. Ten newly hatched *M. persicae* nymphs were placed in the center of each dish. The numbers of aphids on treated and untreated leaves were recorded 24 h after release, with five biological replicates per treatment.

Aphid leaf-choice responses were analyzed using a Bayesian beta-binomial model fitted with the R package brms. For each treatment, the probability of selecting the untreated leaf was estimated from the number of aphids choosing the untreated leaf out of the total number released. Four MCMC chains were run for 8,000 iterations each, including 3,000 warm-up iterations. Posterior means and 95% credible intervals were calculated, and significance was defined as a posterior probability greater than 0.95 that the probability of selecting the untreated leaf exceeded 0.5.

### 2.8 PR1::GUS *Arabidopsis* assay for salicylic acid-related host response

Transgenic *Arabidopsis* thaliana Col-0 plants harboring a PR1::GUS construct were used to assess PR-1 expression induced by culture broth (CB), culture filtrate (CF), and culture suspension (CS) of strain 100-57A. The strain was cultured in R2A medium at 28 °C and 180 rpm for 2 days. The culture was then centrifuged at 13,000 rpm for 10 min at 4 °C. The supernatant was collected as CF, and the cell pellet was resuspended in phosphate-buffered saline to obtain CS. Seeds were sterilized by immersion in 70% ethanol for 30 s, followed by treatment with a bleach solution containing 2% NaOCl and 0.05% Tween-20 for 5 min. After washing with sterile distilled water, the seeds were stored at 4 °C for 2 days. Sterilized seeds were then placed on half-strength MS agar medium containing 2.2 g MS salts, 10 g sucrose, and 8 g phyto agar per liter, supplemented with 50 μg/mL kanamycin, and incubated at 25 °C under a 16 h photoperiod with 80% relative humidity. Twelve-day-old seedlings were treated in 24-well plates with diluted CB, CF, and CS samples at 500-, 1,000-, and 2,000-fold dilutions (2 mL per well), with two seedlings per well. Salicylic acid (SA, 0.1 mM) and R2A were included as positive and negative controls, respectively. The plates were incubated on an orbital shaker at room temperature for 2 days. GUS staining was then performed as previously described (Jefferson et al., 1987). GUS activity was evaluated qualitatively based on blue coloration observed under a microscope (Stemi 508; Carl Zeiss, Germany).

### 2.9 Transcriptome sequencing and analysis

Leaf samples from the R2A control, ASM, and strain 100-57A treatments described in Section 2.6 were used for transcriptome analysis. Samples were collected at 0, 2, 4, 6, 8, and 10 days after treatment, with two independent biological replicates per treatment at each sampling time point. RNA-seq libraries were prepared from 36 leaf samples (three treatments × six sampling time points × two biological replicates). Total RNA was extracted from pepper leaves and used for RNA sequencing by Macrogen (Seoul, Republic of Korea). RNA quality and quantity were evaluated prior to library construction. Illumina TruSeq RNA libraries were prepared following the service provider’s standard protocol, including RNA fragmentation, cDNA synthesis, adapter ligation, index labeling, PCR enrichment, and library quality control. The prepared libraries, with insert sizes of 200–400 bp, were sequenced on an Illumina platform. Raw paired-end reads were quality-filtered using fastp with adapter detection for paired-end reads, poly-G trimming, right-end quality trimming using a mean quality threshold of Q20, and a minimum read length of 50 bp. Per-sample sequencing depth, read filtering statistics, Q30 rates, and Salmon mapping rates are provided in **Supplementary Table 2**.

Trimmed paired-end RNA-seq reads were quantified against the *Capsicum annuum* UCD10Xv1.1 reference transcriptome, derived from the UCD-10X-F1 cultivar assembly (GCF_002878395.1), using Salmon with a decoy-aware selective-alignment approach. Transcript-level abundance estimates were imported into R using tximport and summarized at the gene level based on the reference genome annotation, with countsFromAbundance = “lengthScaledTPM”. Differential expression analysis was performed using DESeq2. For the 0-day comparison, treatment group was used as the main factor. For multi-day intervals, the model included sampling day, treatment group, and their interaction. DESeq2 size-factor normalization was used to account for library-size differences among samples. Log_2_ fold changes were shrinkage-estimated using the ashr method, and genes with adjusted *p* < 0.1 and an absolute shrunken log_2_ fold change > 1 were considered differentially expressed. Gene Ontology over-representation analysis was conducted using clusterProfiler based on the GO annotations of the UCD10Xv1.1 reference genome, and enriched biological process terms with adjusted *p* < 0.1 were retained.

## 3 Results

### 3.1 Aphid treatment altered rhizosphere microbial composition and diversity

**Figure 1:**
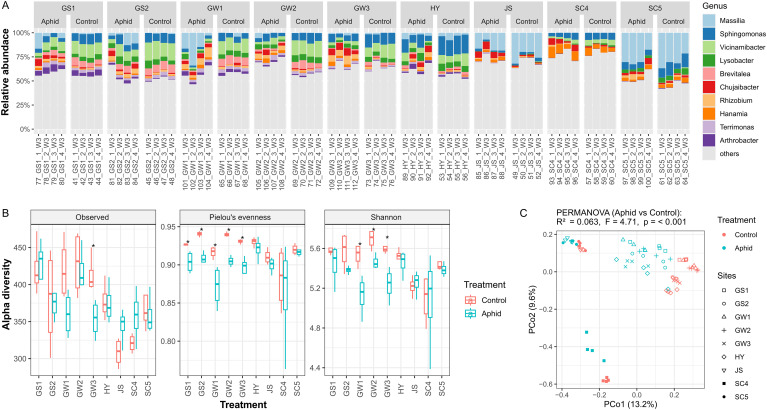
Composition and diversity of the rhizosphere bacterial communities in aphid-treated and control group. **(A)** Relative abundance of the top 10 bacterial genera in the rhizosphere **(B)** Alpha diversity indices including Observed richness, Pielou’s evenness, and Shannon diversity. Each index was compared between aphid-treated and control groups within each site using the Wilcoxon signed-rank test (asterisks indicate p < 0.05). **(C)** Beta diversity visualized by principal coordinates analysis (PCoA) based on Bray-Curtis dissimilarity. Differences between aphid-treated and control group were tested by PERMANOVA.

Rhizosphere bacterial communities were compared between aphid-infested and untreated pepper plants grown in soils collected from nine field sites. At the genus level, the dominant taxa included *Massilia*, *Sphingomonas*, *Vicinamibacter*, *Lysobacter*, and *Brevitalea* (**Figure 1A**). Observed richness did not show a consistent response to aphid infestation across sites. In contrast, Pielou’s evenness tended to decrease in aphid-infested soils at several sites, particularly GS1, GS2, GW1, GW2, and GW3. Shannon diversity exhibited a similar trend, although statistically significant reductions were detected only at GW1, GW2, and GW3 (**Figure 1B**). PERMANOVA based on Bray-Curtis dissimilarity revealed a significant effect of aphid infestation on rhizosphere bacterial community composition, although the explanatory value was relatively low (F = 4.71, R² = 0.063, *p* < 0.001). Consistent with this result, PCoA showed partial separation between aphid-treated and control samples along the first two axes (**Figure 1C**). However, separation was limited at some sites, including JS and SC5.

### 3.2 Soil physicochemical heterogeneity distinguished primary and excluded groups

**Figure 2:**
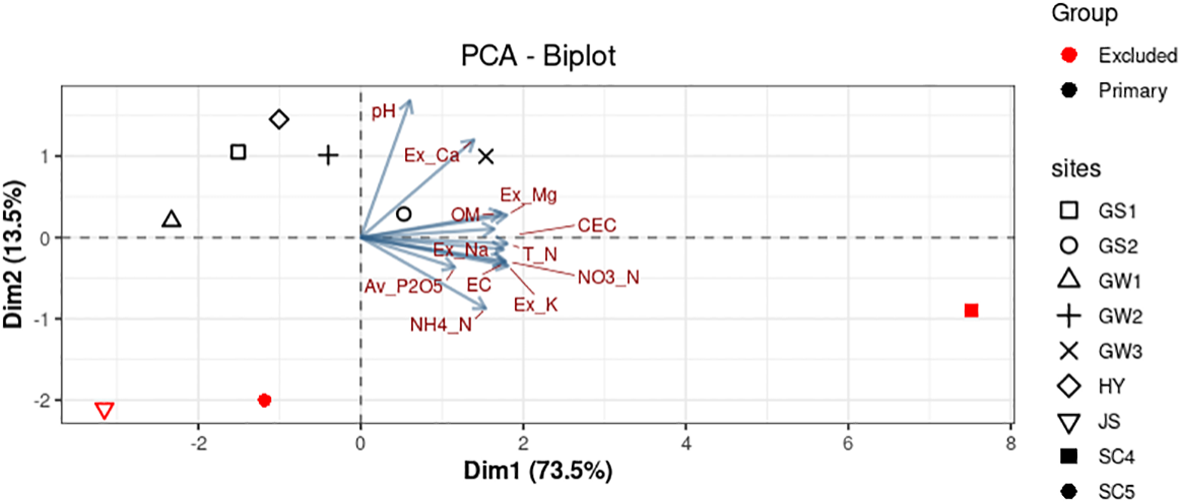
Principal component analysis (PCA) biplot of soil physicochemical properties across nine sampling sites (GS1, GS2, GW1, GW2, GW3, HY, JS, SC4, and SC5). Points represent sites, and arrows indicate the direction and relative contribution of the 12 variables with the highest combined contributions to PC1 and PC2. PC1 and PC2 explained 73.5% and 13.5% of the total variance, respectively. Ex_K, exchangeable K; Ex_Ca, exchangeable Ca; Ex_Mg, exchangeable Mg; Ex_Na, exchangeable Na; NH4_N, ammonium nitrogen; NO3_N, nitrate nitrogen; OM, organic matter; CEC, cation exchange capacity; T_N, total nitrogen; EC, electrical conductivity; Av_P2O5, available phosphate.

The physicochemical properties of the nine field-derived soils are summarized in **Supplementary Table 3**. SC4 showed broadly elevated physicochemical values for most measured variables except pH. In contrast, JS and SC5 were mainly characterized by lower pH than the other sites. To evaluate overall edaphic heterogeneity among the field-derived soils, PCA was performed using the measured soil physicochemical variables (**Figure 2**). PC1 and PC2 explained 73.5% and 13.5% of the total variance, respectively. Together, these two axes explained 87.0% of the total variance (**Figure 2**; **Supplementary Figure 2A**). Variable contribution analysis indicated that PC1 was mainly driven by Ex_K, T_N, Ex_Mg, NO3_N, and EC, whereas PC2 was mainly associated with pH, followed by Ex_Ca and T_N (**Supplementary Figure 2B**). In the PCA score plot, JS, SC4, and SC5 were located outside the 95% confidence ellipse defined by the Primary group and were separated from the main edaphic space. GS1, GS2, GW1, GW2, GW3, and HY were assigned to the Primary group for downstream aphid-responsive microbiome analysis, whereas JS, SC4, and SC5 were assigned to the Excluded group.

### 3.3 Aphid-responsive bacterial taxa in the primary group

**Figure 3:**
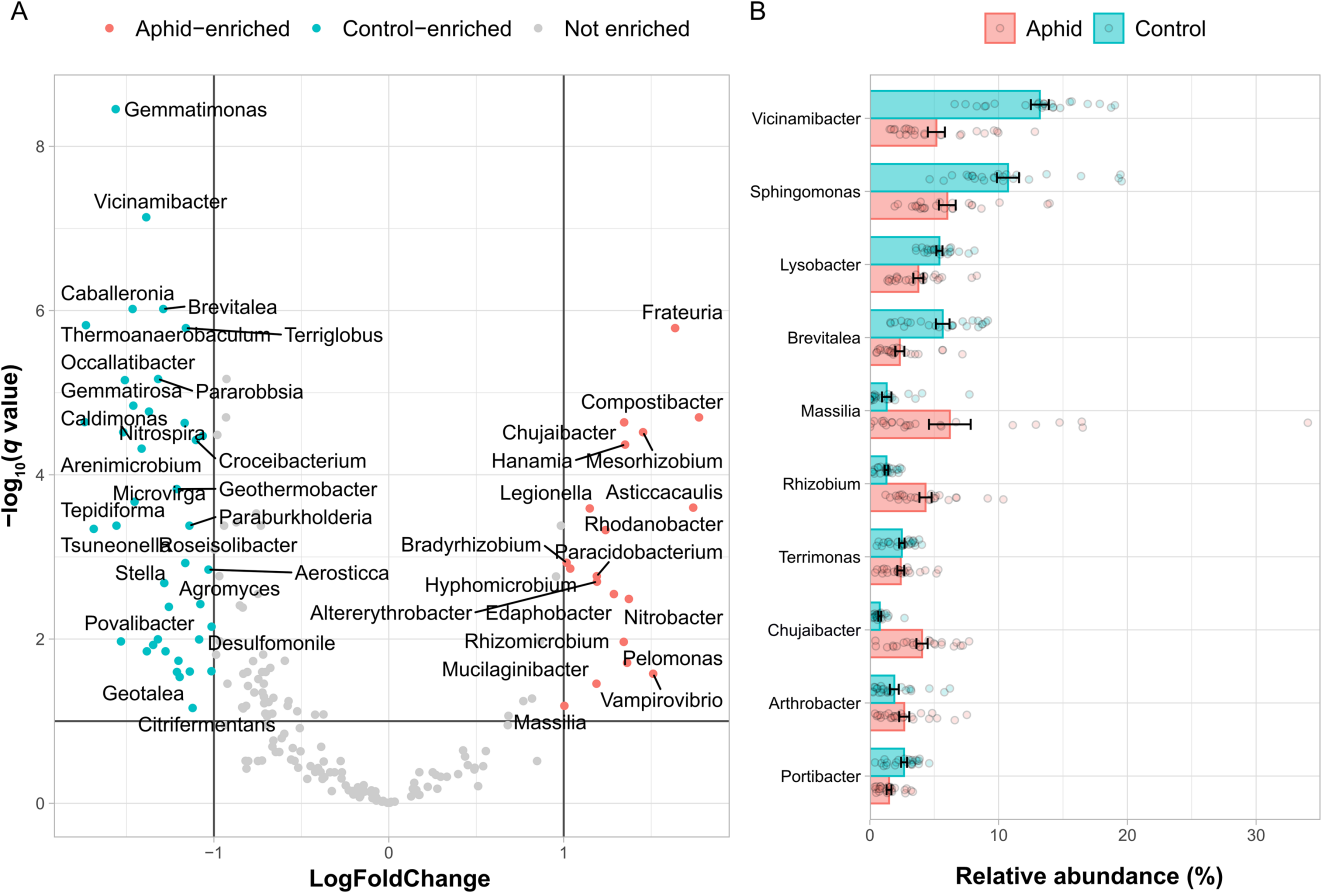
Differential and relative abundance analyses of rhizosphere bacterial genera in the Primary group after exclusion of physicochemically distinct soils. **(A)** Volcano plot comparing aphid-treated and control groups (x-axis: log fold change; y-axis: –log10(q value)). Red points indicate aphid-enriched genera, cyan points indicate control-enriched genera, and gray points indicate non-significant taxa. **(B)** Relative abundance (%) of the 10 most abundant genera in aphid-treated and control groups (points show individual values; error bars indicate SD). Within the top 10 taxa by relative abundance, three genera (Massilia, Rhizobium, and Chujaibacter) were enriched in the aphid-treated group.

Genus-level responses to aphid infestation were evaluated in the Primary group samples (GS1, GS2, GW1, GW2, GW3, and HY) using differential abundance analysis visualized by volcano plots. Multiple genera were differentially abundant between aphid-treated and control rhizosphere soils, indicating that aphid infestation was associated with compositional shifts in specific rhizosphere bacteria (**Figure 3A**). Notably, genera showing higher abundance in control soils appeared to be more numerous than those enriched in aphid-treated soils. To further characterize dominant community members, the top 10 genera based on relative abundance were examined (**Figure 3B**). Among these top genera, *Massilia*, *Rhizobium*, and *Chujaibacter* showed higher relative abundance in aphid-treated soils than in controls, whereas the remaining dominant taxa, including *Vicinamibacter*, *Sphingomonas*, *Lysobacter*, *Brevitalea*, *Terrimonas*, *Arthrobacter*, and *Portibacter* were more abundant in control soils.

### 3.4 Isolation and phylogenetic identification of *Telluria* and *Massilia* strains

**Figure 4:**
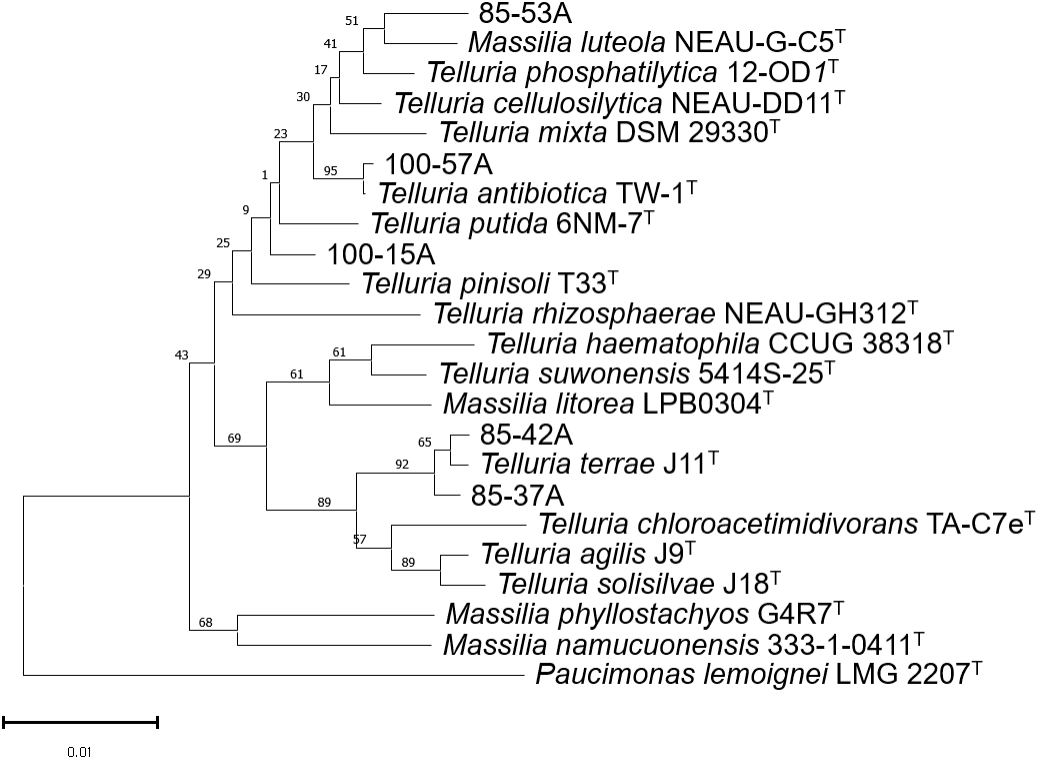
Neighbor-joining phylogenetic tree based on 16S rRNA gene sequences of four Telluria isolates and one Massilia isolate recovered from rhizosphere soil. Paucimonas lemoignei LMG 2207T was used as an outgroup.

Five bacterial strains (85-37A, 85-42A, 85-53A, 100-15A, and 100-57A) were isolated from the rhizosphere soils of aphid-infested pepper plants. Representative colony morphologies of the isolates are shown in **Supplementary Figure 3**. The isolates were then subjected to 16S rRNA gene sequence analysis for taxonomic identification. Strains 85-37A and 85-42A showed the highest sequence similarity to *Telluria terrae* J11, with similarities of 99.48% and 99.47%, respectively. Strain 100-15A was most closely related to *Telluria putida* 6NM-7 with 98.97% similarity, whereas strain 100-57A showed 99.77% similarity to *Telluria antibiotica* TW-1. Strain 85-53A showed 98.74% similarity to *Massilia luteola* NEAU-G-C5. Based on 16S rRNA gene sequence similarity, four isolates were assigned to the genus *Telluria* and one isolate to the genus *Massilia* (**Figure 4**). In the neighbor-joining tree, all five isolates clustered with reference taxa within the family Oxalobacteraceae and were positioned near their closest reference strains. Because 16S rRNA gene analysis alone was insufficient for reliable species-level classification, all isolates were conservatively assigned at the genus level. Among the isolates, strain 100-57A was selected for further analysis because of its aphid suppressive activity. In addition, previously reported whole-genome sequencing and average nucleotide identity (ANI) analyses of strain 100-57A supported its genome-based affiliation with genus *Telluria* (Cho et al., 2025).

### 3.5 Bayesian analysis identified strain 100-57A as the most consistent candidate for aphid suppression

**Figure 5:**
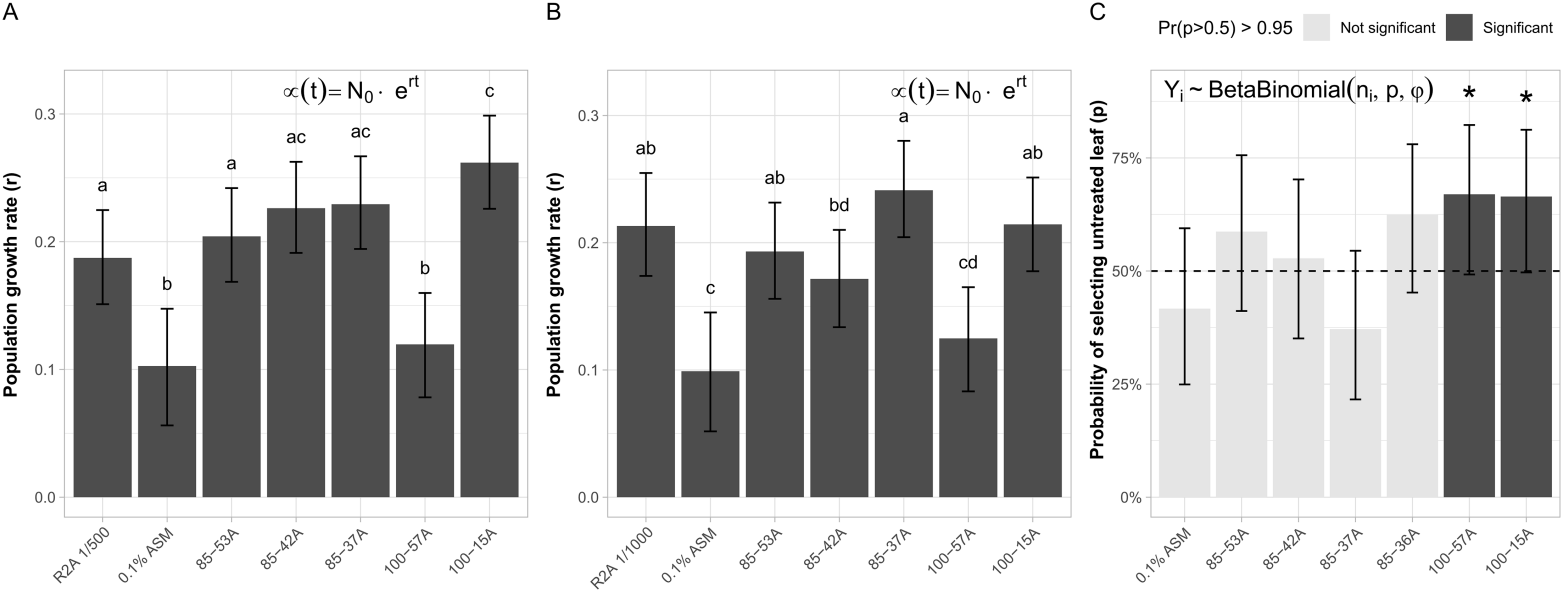
Bayesian model–based estimates of aphid population growth and leaf-choice behavior. **(A)** Posterior summaries of treatment-wise population growth rates for the 500-fold dilutions. **(B)** Posterior summaries of treatment-wise population growth rates for the 1000-fold dilution. **(C)** Posterior summaries of treatment-wise probabilities of selecting the untreated leaf in the dual-choice assay. Bars indicate posterior means and error bars indicate 95% CrI. Compact-letter displays denote treatment groupings derived from posterior pairwise contrasts. For all fitted models, convergence and sampling adequacy were confirmed (R̂ < 1.01, bulk ESS > 1000, tail ESS > 1000).

Bayesian models were used to quantify aphid population growth and dual-choice behavior following microbial treatment. Under both dilution conditions (500- and 1,000-fold), posterior estimates indicated treatment-specific differences in aphid population growth rates (**Figures 5A, B**). At the 500-fold dilution, the posterior mean aphid population growth rate was 0.187 in the negative control and 0.120 in the 100-57A treatment. Similarly, at the 1,000-fold dilution, the estimated growth rates were 0.213 and 0.125 for the negative control and 100-57A treatment, respectively. These values corresponded to 36.2% and 41.5% reductions in aphid population growth in the 100-57A treatment under the 500- and 1,000-fold dilution conditions, respectively. Among the tested strains, only strain 100-57A showed a credibly lower aphid population growth rate than the negative control under both dilution conditions. Posterior pairwise comparisons further indicated that the suppressive effect of strain 100-57A was broadly comparable to that of ASM, although the magnitude of the effect varied depending on dilution level. In the dual-choice assay, only strains 100-57A and 100-15A induced credible aphid avoidance responses, with posterior mean probabilities of selecting untreated leaves of 0.670 and 0.665, respectively (**Figure 5C**). Model diagnostics supported stable inference (R-hat< 1.01, bulk ESS > 1000, tail ESS > 1000; **Supplementary Table 4**). Because strain 100-57A showed the most consistent suppressive effects on aphid population growth across both dilution conditions, it was selected for subsequent analyses. ASVs showing ≥99% identity to the 16S rRNA sequence of strain 100-57A were detected in multiple soils, including GS1, GS2, GW1, JS, and SC5, although their relative abundance varied across sites (**Supplementary Figure 4**).

### 3.6 Strain 100-57A induced time-dependent defense responses and a transcriptomic profile distinct from ASM

**Figure 6:**
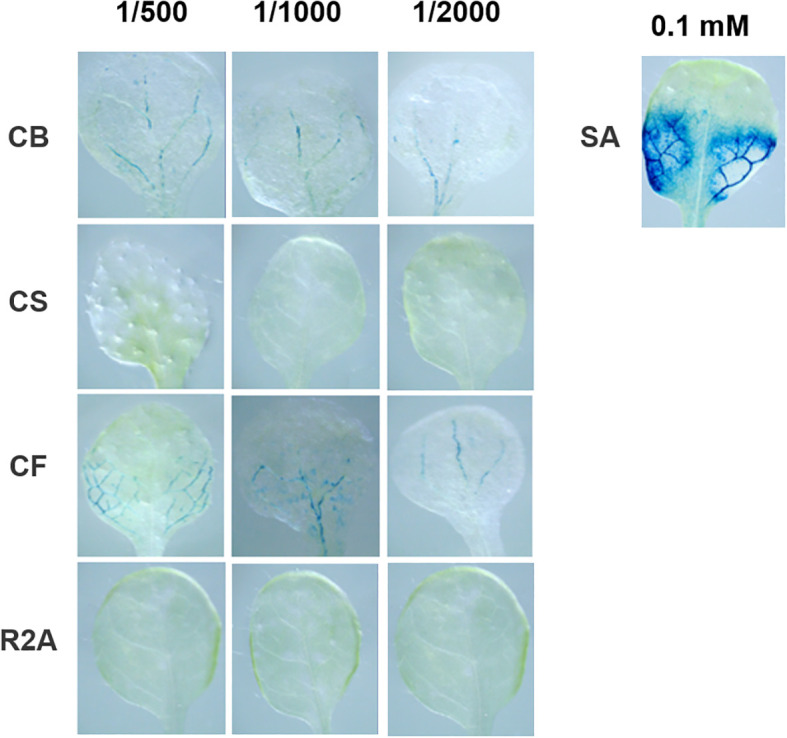
GUS histochemical assay of transgenic *Arabidopsis* seedlings treated with the culture broth (CB), culture suspension (CS), or culture filtrate (CF) of *Telluria* sp. 100-57A. R2A medium and salicylic acid (SA) were used as the negative and positive controls, respectively.

**Figure 7:**
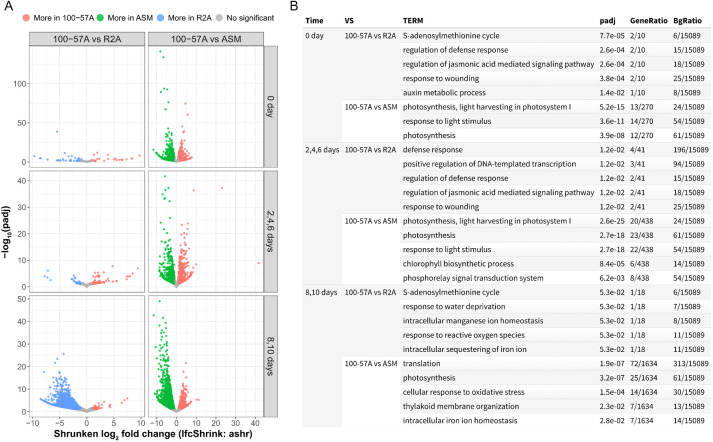
Differential expression and GO Biological Process enrichment in pepper leaves following 100-57A treatment. **(A)** Differential expression across time-point groups. DESeq2 contrasts were performed for pepper leaves treated with the 100-57A strain compared with R2A control and with ASM. Results are summarized by the time-point groups which are 0 day; 2,4,6 days; and 8,10 days. Differentially expressed genes were defined as padj < 0.1 together with an effect-size cutoff of |shrunken log2 fold change| > 1, where log2 fold changes were shrinkage-estimated using ashr. **(B)** GO Biological Process enrichment of genes upregulated in 100-57A. Top 5 enriched GO BP terms among genes classified as “More in 100-57A” (i.e., positive shrunken log2 fold change in the specified contrast) were summarized from the DESeq2 results by Time and contrast (VS). The table reports TERM, padj, and enrichment ratios (GeneRatio, the proportion of differentially expressed genes annotated to the term, and BgRatio, the corresponding proportion in the background gene set).

To examine host defense responses induced by strain 100-57A, GUS histochemical assays were performed using transgenic *Arabidopsis* seedlings (**Figure 6**). The R2A control and CS did not induce detectable GUS staining in PR1::GUS seedlings at any tested dilution. In contrast, CB and CF derived from strain 100-57A induced visible GUS staining at 500-, 1,000-, and 2,000-fold dilutions. Transcriptome analysis was subsequently conducted using pepper leaves treated with strain 100-57A, with contrasts grouped into 0 day, 2, 4, and 6 days, and 8,10 days. Volcano plot analysis showed that the transcriptional response to strain 100-57A varied across time-point groups and according to the reference treatment. Compared with R2A, genes upregulated by 100-57A were most clearly associated with defense-related responses in the 0 day group and the 2, 4, and 6 day group. This pattern was not maintained in the 8 and 10 day group. Consistent with these results, GO Biological Process enrichment analysis of upregulated genes revealed significant enrichment of defense-related, jasmonic acid signaling-related, and wound-response-related categories. These categories were enriched in the 0 day group and the 2 to 6 day group, but not in the 8 to 10 day group (**Figure 7B**). In comparison with ASM, strain 100-57A showed distinct transcriptional response patterns across all sampled time-point groups. Genes upregulated by strain 100-57A relative to ASM were repeatedly enriched for photosynthesis- and light-related categories, including photosynthesis, response to light stimulus, and chlorophyll biosynthetic process.

## 4 Discussion

Biological control is an important strategy for sustainable crop protection because it can reduce dependence on chemical inputs while promoting ecological resilience in agroecosystems (Bale et al., 2007; Schaffner et al., 2024). Considerable attention has been given to microbiome-mediated protection, whereby plants under biotic stress can recruit beneficial microorganisms, a response often referred to as a “cry for help” (Berendsen et al., 2012; Rolfe et al., 2019). Although this phenomenon has been studied primarily in plant–pathogen interactions, growing evidence suggests that herbivore attack can also alter rhizosphere microbial assembly (Yang et al., 2011). Consistent with this concept, aphid infestation in the present study was accompanied by changes in the taxonomic composition of rhizosphere bacterial communities, whereas alpha diversity responses varied among sites. Observed richness did not show a consistent decrease after aphid infestation, indicating that aphid treatment did not broadly reduce the number of bacterial taxa present in the rhizosphere. In contrast, Pielou’s evenness tended to decrease in several aphid-treated soils, and Shannon diversity was significantly reduced at GW1, GW2, and GW3. Because Shannon diversity reflects both richness and evenness, these results suggest that the observed reductions were primarily driven by changes in community structure rather than by a loss of bacterial taxa. This pattern suggests that aphid infestation shifted the balance of the rhizosphere bacterial community by increasing the relative dominance of specific bacterial groups. Such selective enrichment may represent an ecologically important component of plant defense, as recruited microorganisms can subsequently influence host resistance and stress adaptation (Berendsen et al., 2018; Carrión et al., 2019). Aphid infestation accounted for only a small fraction of the total community variation in the present study (R² = 0.063). However, this effect size was comparable to previous reports on aphid-induced rhizosphere microbiome shifts. For example, high aphid infestation explained approximately 6 to 7% of rhizosphere bacterial community variation in tomato in a soil-dependent manner (French et al., 2021). In cabbage, aphid herbivory explained 5.7% of Bray-Curtis variation, although the effect was not statistically significant (O’Brien et al., 2018). These findings suggest that aphid effects on rhizosphere bacterial community composition are generally modest and context dependent, but can still be detected across different soil backgrounds.

Soil heterogeneity among sites was considered because it could influence the interpretation of aphid-associated microbiome responses. PCA of soil physicochemical properties revealed substantial edaphic variation among the nine field-derived soils, with SC4, SC5, and JS clearly separated from the remaining sites. This separation was driven primarily by differences in pH, EC, nitrogen status, and exchangeable cations (**Supplementary Figure 2**). Consistent with these patterns, bacterial community ordination also showed distinct clustering of these soils. This result suggests that soil physicochemical properties contributed strongly to rhizosphere bacterial community assembly, consistent with previous studies highlighting the dominant role of soil conditions in determining rhizosphere microbiome structure (Fierer and Jackson, 2006; Berg and Smalla, 2009). In aphid-associated rhizosphere studies, aphid effects on bacterial diversity and community composition have also been shown to depend on soil origin, indicating that soil background can strongly influence the apparent magnitude and direction of herbivore-induced microbiome responses (French et al., 2021). Given this clear physicochemical separation, SC4, SC5, and JS were assigned to the edaphically distinct group, whereas the remaining six sites were retained as the Primary group to reduce pronounced edaphic confounding in the downstream aphid-responsive microbiome analysis.

Because differential abundance analysis was used as a discovery step to identify candidate taxa for isolate-based validation, an exploratory FDR threshold was adopted to reduce false negatives (Benjamini and Hochberg, 1995; Goeman and Solari, 2011). Among the aphid-enriched genera, the *Massilia*-associated amplicon signal was selected as a primary candidate because it showed a consistent positive response to aphid treatment and represented one of the most abundant signals in the community dataset. To follow up this community-level signal, isolate-based screening from aphid-treated rhizosphere soils recovered strains assigned to both *Massilia* and *Telluria*. The difference between the amplicon-level signal and isolate-level assignment is taxonomically plausible within Oxalobacteraceae. Several species previously classified as *Massilia* have been reassigned to *Telluria* and related genera (Bowman, 2023). These taxonomic changes reflect the close phylogenetic relationships among members of this clade. Therefore, the *Massilia*-enriched amplicon signal may represent a broader *Massilia* and *Telluria*-related lineage response rather than the expansion of a single clearly delimited genus.

Previous studies have reported that aphid infestation can alter belowground microbial communities (French et al., 2021), and recruit beneficial rhizosphere bacteria while priming plant immunity in pepper (Lee et al., 2012). However, whether aphid-responsive taxa can be translated into functional biocontrol candidates remains largely unexplored. In the present study, five bacterial isolates recovered from aphid-treated rhizosphere soils were evaluated for aphid-suppressive activity, and strain 100-57A showed the most consistent effects in both aphid population growth and choice assays. These results indicate that aphid-associated enrichment did not necessarily translate into equivalent suppressive activity among related isolates. Instead, microbiome-derived community signals are useful for candidate discovery but insufficient to infer biological efficacy without direct validation of individual strains (Fitzpatrick et al., 2020; Fonseca-Garcia et al., 2025). The superior performance of strain 100-57A further suggests that its suppressive activity was linked to strain-specific functional traits rather than enrichment patterns alone. Although the mechanisms underlying the activity of strain 100-57A remain unclear, previous studies suggest that microbial metabolites and elicitors can contribute to aphid suppression and induced resistance. In pepper plants, a synthetic community composed of Gram-positive bacteria produced 1-nonanol, and both the community treatment and exogenous 1-nonanol reduced aphid infestation (Lee et al., 2025). Likewise, exogenous applications of 3-pentanol and 2-butanone reduced aphid infestation in cucumber under field conditions (Song and Ryu, 2013). In addition to VOCs, non-volatile metabolites and secreted elicitors produced by beneficial bacteria can activate hormone-dependent defense responses and prime systemic resistance against pathogens and insect herbivores (Ongena et al., 2007; Pangesti et al., 2016; Pršić and Ongena, 2020). Consistent with this possibility, the culture broth and culture filtrate of strain 100-57A induced PR1::GUS activity, whereas the culture suspension did not. Therefore, extracellular metabolites or secreted elicitors may contribute to the defense-related host responses observed in this study. However, VOC production and specific active metabolites were not identified here. Further metabolite profiling and functional validation will be needed to clarify the underlying mechanisms.

Host response assays further suggested that aphid suppression by strain 100-57A was associated with microbe-induced defense priming before aphid infestation. This interpretation is consistent with previous studies showing that beneficial rhizosphere microorganisms can prime systemic resistance against pathogens and insect herbivores through hormone-dependent defense regulation (Pieterse et al., 2014; Pangesti et al., 2016; Yang et al., 2022). PR1::GUS activation indicated the involvement of SA-associated defense responses. However, transcriptome analysis showed stronger enrichment of general defense regulation, jasmonic acid signaling, and wound-response categories than a distinct SA-dominated transcriptional signature. This mixed pattern may reflect both host-dependent defense regulation and hormone crosstalk. The PR1::GUS assay was conducted in *Arabidopsis*, whereas transcriptome analysis was conducted in pepper. Therefore, the SA-associated reporter response and the broader pepper transcriptional profile are not expected to match completely. Host-dependent variation in rhizobacteria-induced systemic resistance has been reported previously (van Loon et al., 1998), and SA- and JA-associated pathways can also interact during plant immune regulation (Van der Does et al., 2013). Notably, these defense-associated categories were enriched during the early response period (0 to 6 days) but not at later time points (8 to 10 days), supporting a transient inducible response rather than prolonged constitutive activation. Such a transient response is important because constitutive defense activation can impose growth costs, whereas priming allows plants to maintain a defense-ready state without continuous activation of costly defense responses (van Hulten et al., 2006; Pieterse et al., 2014; Karasov et al., 2017). The transcriptional response induced by strain 100-57A also differed from that of ASM treatment. Because ASM is a functional analog of salicylic acid and a well-established inducer of systemic acquired resistance (SAR), a response driven primarily by canonical SA signaling would be expected to resemble ASM more closely (Lawton et al., 1996; Tripathi et al., 2019). Instead, strain 100-57A induced greater enrichment of photosynthesis- and chloroplast-related categories, suggesting that its defense-associated effects may be achieved through mechanisms distinct from those of ASM treatment. Although the physiological significance of these transcriptional patterns requires further validation through direct measurements of photosynthetic performance and plant growth, the results suggest that strain 100-57A may promote aphid resistance while maintaining host physiological functions.

Despite these promising results, several practical challenges remain before strain 100-57A can be developed as an aphid management agent. Its efficacy in edaphically distinct soils was not evaluated in the present study. This includes the low-pH soils and high-EC, nutrient-enriched soils assigned to the Excluded group. Additional greenhouse and field tests across contrasting soil environments are therefore needed. Such edaphic conditions may affect strain survival, root colonization, and the consistency of aphid suppression. Application timing and dose should also be optimized because defense priming may depend on the interval between microbial treatment and aphid infestation. In addition, formulation studies are needed to improve shelf life, delivery efficiency, and stability after application. Compatibility with existing pest management practices, including reduced insecticide programs, should also be evaluated. Finally, metabolite profiling, colonization assays, and functional validation will be required to identify the microbial traits responsible for aphid suppression. These steps will help determine whether strain 100-57A can be translated from a microbiome-derived candidate into a reliable biocontrol product.

## 5 Conclusion

This study demonstrated that aphid infestation was associated with restructuring of the pepper rhizosphere microbiome and enabled the identification of bacterial taxa linked to aphid-responsive community shifts. Using a microbiome-guided approach that combined community profiling, isolate recovery, and functional validation, we identified *Telluria* sp. 100-57A as a promising candidate for aphid suppression. This strain consistently reduced aphid population growth, altered aphid host preference, and induced defense-associated host responses prior to aphid infestation. Transcriptome analysis further indicated that strain 100-57A triggered a defense-related response distinct from that induced by ASM treatment while maintaining enrichment of photosynthesis-related functions. These findings demonstrate the value of microbiome-informed strategies for discovering beneficial microorganisms associated with pest suppression and highlight aphid-responsive rhizosphere microbiomes as a potential reservoir of biocontrol candidates. Further studies are needed to identify the microbial determinants underlying the activity of strain 100-57A and to evaluate its performance under greenhouse and field conditions. In addition, formulation development and environmental stability assessments will be required to support its practical application for sustainable aphid management in pepper.

## Data Availability

The raw sequencing datasets generated in this study, including full-length 16S rRNA gene amplicon sequencing data and RNA-seq data, are available in the NCBI BioProject repository under accession number PRJNA1431720.
