# Supplementary material for: Integrative microbiome and transcriptome analyses reveal Telluria sp. 100-57A as a candidate for aphid suppression in pepper
Source: Front Plant Sci. 2026 Jul 6;17:1861231. doi: 10.3389/fpls.2026.1861231 (PMC13381450; doi:10.3389/fpls.2026.1861231)
Supplement: Supplementary file 2 [file Table2.docx]

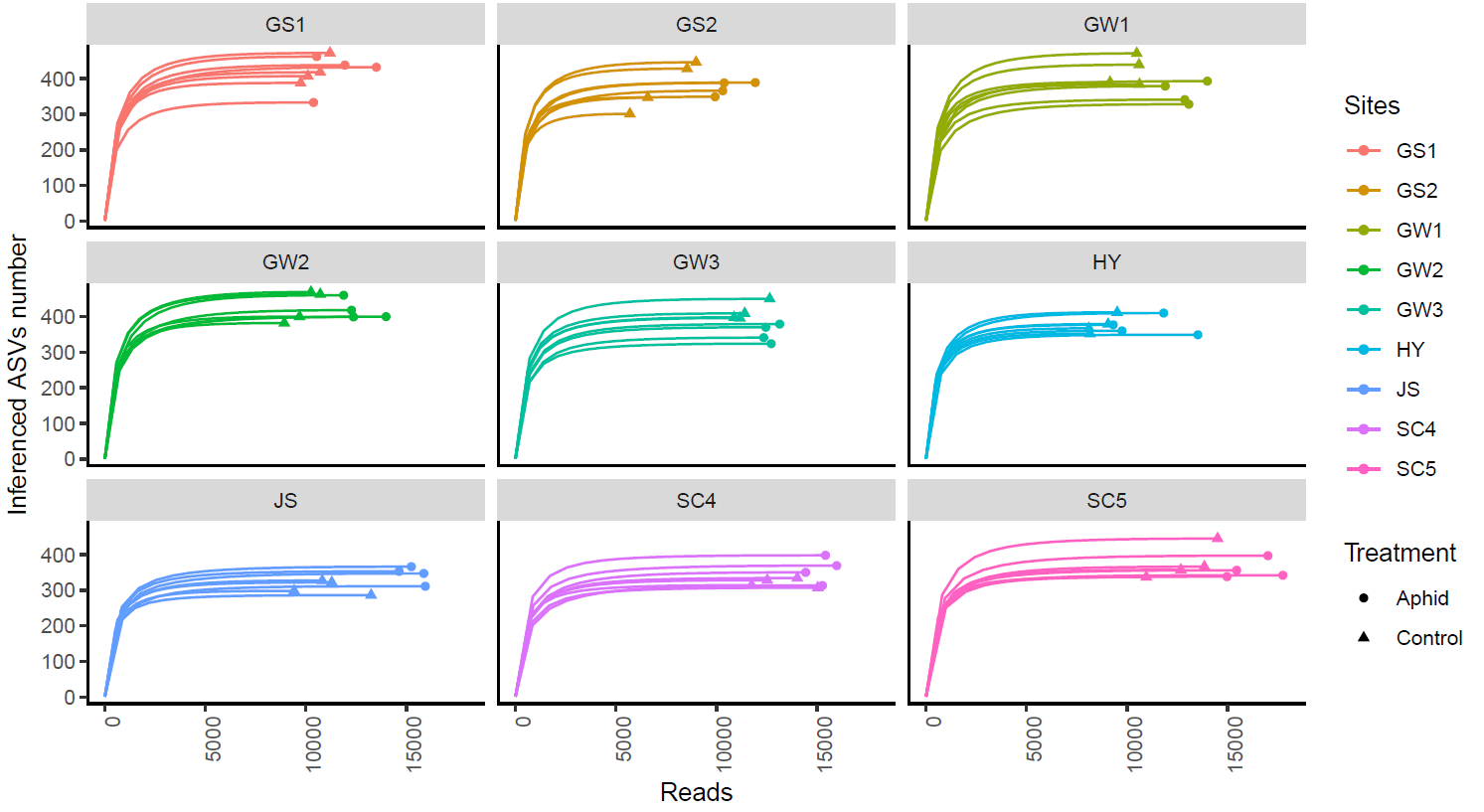
**Figure S1.** Rarefaction curves showing sequencing depth sufficiency for rhizosphere bacterial community analysis.


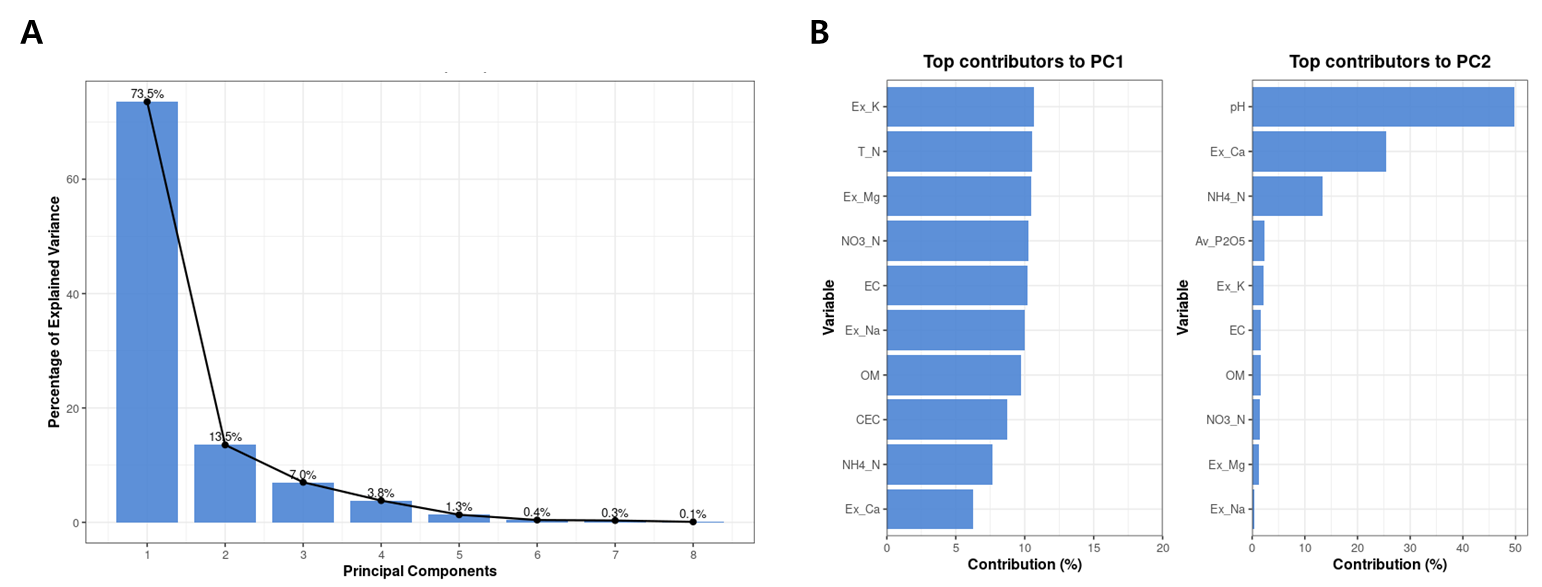


**Figure S2.** Explained variance and variable contributions in the principal component analysis of soil physicochemical properties. (A) Scree plot showing the percentage of variance explained by each principal component (PC). PC1 and PC2 explained 73.5% and 13.5% of the total variance, respectively (cumulative 87.0%). (B) Bar plots showing the top contributing variables for PC1 and PC2. PC1 was primarily associated with Ex_K, T_N, Ex_Mg, NO3_N, and EC, whereas PC2 was primarily associated with pH, followed by Ex_Ca and NH4_N. Contributions are presented as percentages based on PCA loadings.

**
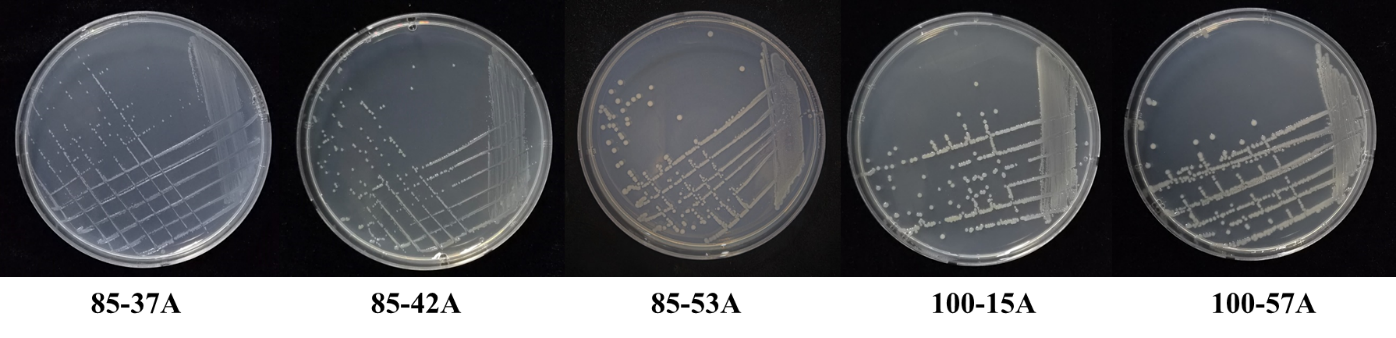
Figure S3.** Representative colony morphology of bacterial isolates from aphid-infested pepper rhizosphere soils. Each strain was grown on R2A agar plates 28 °C for 3 days.


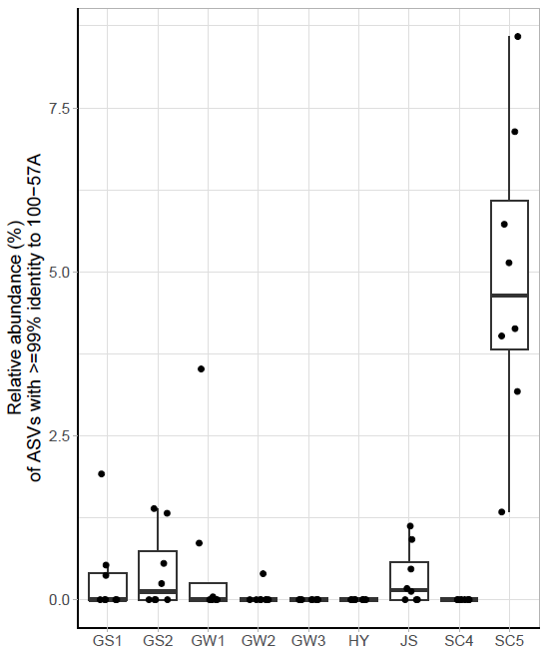


**Figure S4.** Relative abundance (%) of ASVs showing ≥99% sequence identity to the full-length 16S rRNA gene sequence of *Telluria* sp. 100-57A across soils.

|  | **GS1** | **GS2** | **GW1** | **GW2** | **GW3** | **HY** | **JS** | **SC4** | **SC5** |
| --- | --- | --- | --- | --- | --- | --- | --- | --- | --- |
| **Ex-K (cmolc/kg)** | 0.3(± 0.0) e | 2.0 (± 0.1) b | 0.7 (± 0.0) d | 1.2 (± 0.0) c | 1.7 (± 0.1) b | 0.8 (± 0.0) cd | 0.5 (± 0.0) de | 5.9 (± 0.4) a | 1.1 (± 0.0) c |
| **Ex-Ca (cmolc/kg)** | 8.8 (± 0.3) c | 7.8 (± 0.1) d | 5.3 (± 0.0) e | 8.1 (± 0.2) cd | 9.7 (± 0.1) b | 9.7 (± 0.2) b | 1.9 (± 0.0) g | 12.1 (± 0.7) a | 3.5 (± 0.1) f |
| **Ex-Mg (cmolc/kg)** | 2.1 (± 0.1) d | 2.3 (± 0.1) cd | 1.4 (± 0.0) e | 2.8 (± 0.1) c | 4.0 (± 0.0) b | 1.4 (± 0.1) e | 0.5 (± 0.0) f | 6.5 (± 0.5) a | 1.1 (± 0.0) e |
| **Ex-Na (cmolc/kg)** | 0.5 (± 0.0) c | 0.3 (± 0.0) d | 0.2 (± 0.0) d | 0.2 (± 0.0) d | 0.7 (± 0.0) b | 0.1 (± 0.0) d | 0.1 (± 0.0) de | 2.0 (± 0.2) a | 0.1 (± 0.0) d |
| **NH4-N (mg/kg)** | 4.6 (± 0.8) cde | 5.6 (± 0.1) cd | 1.7 (± 0.3) e | 2.5 (± 0.6) e | 7.3 (± 0.4) c | 4.0 (± 0.3) de | 4.3 (± 0.2) de | 66.3 (± 0.6) a | 31.2 (± 2.8) b |
| **NO3-N (mg/kg)** | 20.1 (± 1.5) g | 85.9 (± 4.0) c | 52.7 (± 3.7) e | 30.5 (± 1.2) f | 240.3 (± 7.2) b | 6.6 (± 0.4) h | 10.9 (± 0.3) h | 502.9 (± 1.3) a | 67.4 (± 1.2) d |
| **pH(1:5)** | 6.5 (± 0.0) d | 6.4 (± 0.0) e | 6.8 (± 0.0) c | 6.9 (± 0.0) c | 7.0 (± 0.0) b | 7.4 (± 0.1) a | 4.5 (± 0.0) g | 6.6 (± 0.0) d | 5.1 (± 0.1) f |
| **OM (g/kg)** | 26.7 (± 0.6) e | 46.6 (± 1.3) b | 15.0 (± 0.7) f | 40.0 (± 2.2) c | 47.8 (± 1.5) b | 31.3 (± 0.6) d | 15.9 (± 0.8) f | 64.5 (± 1.0) a | 29.5 (± 0.5) de |
| **CEC (cmolc/kg)** | 19.3 (± 0.4) c | 21.7 (± 0.2) b | 10.5 (± 0.3) f | 19.2 (± 1.5) c | 18.9 (± 0.6) c | 15.0 (± 0.8) de | 13.2 (± 0.8) e | 27.7 (± 0.6) a | 16.5 (± 0.2) d |
| **T-N (%)** | 0.1 (± 0.0) e | 0.3 (± 0.0) b | 0.1 (± 0.0) f | 0.2 (± 0.0) d | 0.3 (± 0.0) c | 0.2 (± 0.0) e | 0.1 (± 0.0) f | 0.5 (± 0.0) a | 0.2 (± 0.0) e |
| **EC (dS/m)** | 1.3 (± 0.1) c | 1.3 (± 0.0) c | 0.8 (± 0.0) d | 0.5 (± 0.0) e | 4.3 (± 0.1) b | 0.4 (± 0.0) ef | 0.3 (± 0.0) f | 14.7 (± 0.1) a | 0.9 (± 0.0) d |
| **Av-P2O5 (mg/kg)** | 197.4 (± 6.1) f | 1328.5 (± 78.7) b | 993.5 (± 78.8) de | 1109.1 (± 143.1) cd | 1129 (± 19.8) cd | 1251.5 (± 20.1) bc | 809.9 (± 38.1) e | 1621.2 (± 19.2) a | 1274.5 (± 83.5) bc |

**Table S3.** Soil physicochemical properties of soils used in this study.

* Values are means ± SE of three biological replicates. Differing letters indicate significant differences by Tukey’s Honest Significant Differences at *p* < 0.05 compared across the three soil
